# Airborne Aluminum as an Underestimated Source of Human Exposure: Quantification of Aluminum in 24 Human Tissue Types Reveals High Aluminum Concentrations in Lung and Hilar Lymph Node Tissues

**DOI:** 10.1021/acs.est.4c01910

**Published:** 2024-06-18

**Authors:** Clara Ganhör, Lukas Mayr, Julia Zolles, Marion Almeder, Matin Kazemi, Markus Mandl, Christian Wechselberger, Dave Bandke, Sarah Theiner, Christian Doppler, Andreas Schweikert, Marina Müller, Špela Puh, Michaela Kotnik, Rupert Langer, Gunda Koellensperger, David Bernhard

**Affiliations:** † Division of Pathophysiology, Institute of Physiology and Pathophysiology, Medical Faculty, Johannes Kepler University, Linz 4020, Austria; ‡ Institute of Analytical Chemistry, Faculty of Chemistry, University of Vienna, Vienna 1090, Austria; § Institute of Clinical Pathology and Molecular Pathology, Kepler University Hospital and Johannes Kepler University, Linz 4020, Austria; ∥ Clinical Research Institute for Cardiovascular and Metabolic Diseases, Medical Faculty, Johannes Kepler University, Linz 4020, Austria

**Keywords:** aluminum, ICP–MS, lumogallion, air pollution, PM_10_–PM_2.5_, tissue distribution

## Abstract

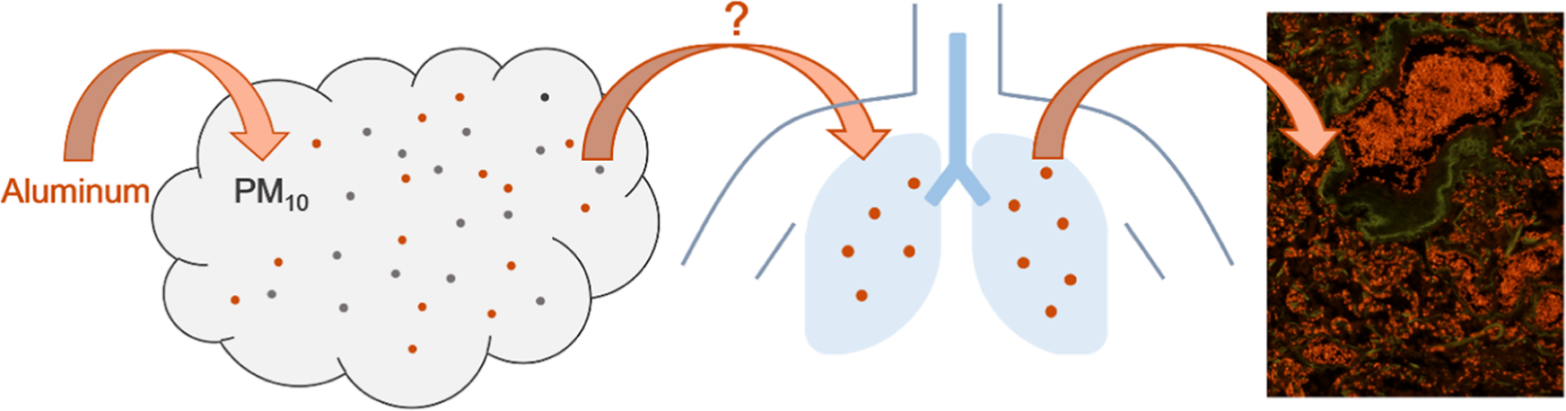

Aluminum (Al) is the most abundant metal in the earth’s crust, and humans are exposed to Al through sources like food, cosmetics, and medication. So far, no comprehensive data on the Al distribution between and within human tissues were reported. We measured Al concentrations in 24 different tissue types of 8 autopsied patients using ICP–MS/MS (inductively coupled plasma–tandem mass spectrometry) under cleanroom conditions and found surprisingly high concentrations in both the upper and inferior lobes of the lung and hilar lymph nodes. Al/Si ratios in lung and hilar lymph node samples of 12 additional patients were similar to the ratios reported in urban fine dust. Histological analyses using lumogallion staining showed Al in lung erythrocytes and macrophages, indicating the uptake of airborne Al in the bloodstream. Furthermore, Al was continuously found in PM_2.5_ and PM_10_ fine dust particles over 7 years in Upper Austria, Austria. According to our findings, air pollution needs to be reconsidered as a major Al source for humans and the environment.

## Abstract 2

Little is known about the distribution of aluminum within humans. In this study, human lung tissues revealed surprisingly high Al concentrations, identifying airborne Al as a potentially underestimated source of human Al.

## 1 Introduction

Aluminum (Al) is the third most common element and the most abundant metal in the earth’s crust, but it is the only metal with no known essential biological function in any living species.^1,2^ Through its use in multiple fields, for example, the automotive industry, food packaging and additives, vaccination adjuvants, and wastewater treatment plants, human exposure to Al has increased significantly since the rise of industrialization.^3−9^

Food is known to be the most important source of human Al, mainly due to its use in food additives and food colors.^10^ Al in antiperspirants has long been discussed as potentially harmful.^11,12^ In 2020, a dermal Al bioavailability of 0.00052% was calculated, and thus Al is considered safe to use in antiperspirants.^13^ Other sources of human Al exposure are cosmetics and air pollution, especially particulate matter (PM) with diameters smaller than 10 μm (PM_10_) or 2.5 μm (PM_2.5_).^14−17^

The oral bioavailability of Al is 0.1% but can vary depending on the Al species one is exposed to.^18−21^ Serum Al levels are 0.06 μM, with about 90% of serum Al being bound by transferrin, but this bond is rather weak.^22−25^ Al injected intravenously is excreted quickly but not completely. Thus, transfer to tissues is very quick. Al is excreted via urine, where an average concentration of 0.33 μM is reported in the literature.^25^ The highest tissue Al concentrations are observed in the bone, liver, and kidney.^19,24^

An in vivo study orally administering Al citrate to mice performed by Quartley et al. showed that the concentrations in soft tissues initially increased and then decreased, while bone levels continually increased and the brain remained unaffected.^26^ Another study performed by Pogue et al. investigated the distribution of orally administered Al from Al sulfate in mice in 25 tissue types after up to five months. The highest accumulation was found in the brain tissue/retina, breast tissue, and ovaries.^27^

In a toxicokinetic model of the distribution of Al citrate and Al chloride in human males after intravenous administration by Hethey et al., under-investigated tissues like lungs, interstitial body fluids, etc. were considered as “rest of the body” but contained the highest Al levels shortly after the initial Al exposure. After 150 weeks, urine and bones have the highest Al fraction, followed by brain and liver.^28^ Al can cross the blood–brain barrier and influences essential brain processes like synaptic transmission, axonal transport, and neurotransmitter synthesis. Furthermore, some reports link Al to neurodegeneration, but this potential connection is still not fully understood.^29,30^ Case reports of individuals exposed to elevated Al levels in drinking water due to an accidental discharge of aluminum sulfate in Camelford, UK, who later suffered from neurodegeneration, showed elevated Al levels in brain tissues.^31^

Similar to the study by Hethey et al., most previous investigations toward Al distribution between human tissues have studied a very limited number of tissue types. Thus, the aim of our study is to shed light on the Al concentration in a much larger set of tissue types, including under-investigated tissues like oral mucosa, lymph nodes, lungs, blood vessels, and the gastrointestinal system. In total, we measured the Al concentrations of 24 tissue types obtained from autopsies of eight patients (4 female and 4 male) using inductively coupled plasma–tandem mass spectrometry (ICP–MS/MS) under cleanroom conditions and histologically investigated the intratissue distribution of Al in selected tissues using lumogallion, an Al-specific fluorescent dye. After identifying lung and hilar lymph node tissues to contain some of the highest concentrations, we collected samples of these tissues from 12 additional patients (8 female and 4 male) and measured both Al and silicon (Si) using inductively coupled plasma–sector field mass spectrometry (ICP–SFMS) to investigate if fine dust is the source of Al found in these tissues because the Al/Si ratio of fine dust is well defined. Furthermore, Al was found in all samples of PM_2.5_ and PM_10_ taken at 18 different measurement sites in Upper Austria, Austria, for up to seven years. To the best of our knowledge, this is the first study including such a large number of patients and tissue types, which now allows us to paint an almost complete picture of the Al distribution within the human body and which surprisingly indicates that airborne Al has been overlooked until now.

## 2 Materials and Methods

### 2.1 Ethics Approval

This study was approved by the Ethics Committee of Johannes Kepler University Linz (EK Nr: 1267/2021).

### 2.2 Sample Collection

The following 24 tissue types were collected from 8 patients (4 female and 4 male) who were autopsied at Kepler University Hospital Linz: fingernails, abdominal skin, oral mucosa, cartilage, trachea, right upper lobe of the lung, right inferior lobe of the lung, hilar lymph nodes, diaphragm, left ventricle of the heart, vena cava, thoracic aorta, intraabdominal fat, stomach, duodenum, ileum, colon, pancreas, kidneys, spleen, liver, urinary bladder, bones, and the psoas major muscle. Samples of right upper and inferior lobes of the lung as well as hilar lymph node tissues of additional 12 patients were collected (8 female and 4 male) for Al and Si measurements.

All tubes for sample collection and storage were immersed in 10% HNO_3_ Suprapur (diluted from HNO_3_ 65% Suprapur, Supelco, VWR, Vienna, Austria) for 24 h, followed by immersion in 1% HNO_3_ Suprapur for 24 h, and then thoroughly rinsed with Milli-Q water. The time between death and autopsy was between 0 and 3 days. The following patient data was obtained: date of birth and death, sex, height, weight, place of residence, pre-existing conditions, smoking status, and medication.

### 2.3 ICP–MS Sample Preparation and Analysis for Al Tissue Distribution Measurements

A total of 191 tissue samples were measured for the first 8 patients. Approximately 300 mg of each sample was aliquoted in a representative manner and weighed in clean tubes using ceramic utensils. Samples were stored at −80 °C until further processing. For detailed information on chemicals and reagents, sample preparation, and ICP–MS/MS measurements, see Supporting Information S1 and Table S1. In short, samples were transferred into microwave extraction vessels together with HNO_3_, and for patients 6–8 with H_2_O_2_, for microwave extraction. Samples were diluted to reach a final HNO_3_ concentration of 3%. Al quantification was performed using ICP–MS/MS. To rule out spectral overlaps, Al was measured as AlO^+^. Quantification was performed by a 9-point matrix-matched external calibration. The mean Al concentration found in the certified plasma reference material BCR-639 was 212 ± 12 μg/L (mean ± standard deviation), which is in the certified range of 194 ± 14 μg/L. The analytical lower limit of quantification (LLOQ) varied between 0.5 and 1 μg/L and was determined as ten times the standard deviation of blank extractions.

### 2.4 ICP–MS Sample Preparation and Analysis for Al and Si Measurements

36 samples were collected from 12 patients, and approximately 300 mg of each sample was aliquoted and weighed as described above. Samples were stored at −80 °C until further processing. For detailed information on chemicals and reagents, sample preparation, and ICP–SFMS measurements, see Supporting Information S2 and Table S2. In short, approximately 300 mg of each sample was weighed in vessels, and HNO_3_, H_2_O_2_, and HF were added for the first microwave extraction step. Then, for complexation of HF, H_3_BO_3_ was added, followed by a second microwave extraction step. Samples were diluted to fit in the working range of 0.1–100 μg L^–1^ and were spiked with an internal standard. Al and Si quantification was performed using ICP–SFMS with high mass resolution to allow for interference-free measurements. Quantification was performed by an 8-point matrix-matched external calibration. For the 10 mg kg^–1^ spike experiment, Al and Si recovery were 94 and 95%, respectively. For the 200 mg kg^–1^ spike experiment, Al and Si recovery were 94 and 113%, respectively.

### 2.5 Histology

For histological analysis, fresh tissues were fixed in 4.5% formaldehyde (Merck, Vienna, Austria) for 48 h and processed using a KOS Rapid Microwave Labstation (Milestone, Bergamo, Italy) following the manufacturer’s protocol using absolute ethanol, isopropanol (both VWR, Vienna, Austria), and paraffin (Surgipath Paraplast Plus, Leica Biosystems, Vienna, Austria). For patients 5 and 7, no histological samples were produced due to a necessary freeze–thaw of samples between autopsy and sample preparation, which could result in changes in aluminum (Al) distribution within tissues. This freeze–thaw process was required due to quarantine measures of personnel.

5 μm sections of tissue samples were prepared using a Leica RM2245 microtome (Leica Biosystems, Vienna, Austria) and stained with lumogallion (TCI Germany, Eschborn, Germany) as described elsewhere.^32^ In brief, slides were dewaxed in Xylene Substitute (Merck, Vienna, Austria) twice for 5 min, rehydrated using an ethanol (VWR, Vienna, Austria) gradient (100, 95, 90, 70, 50, and 30% for 30 s each), washed with water for 1 min, and stained in 1 mM lumogallion in 50 mM piperazine-*N*,*N*′-bis(2-ethanesulfonic acid) (PIPES, Merck, Vienna, Austria) buffer at pH 7.4 for 45 min, followed by washing six times in 50 mM PIPES and once with water. Slides were mounted with ProLong Diamond Antifade Mountant (Molecular Probes, Fisher Scientific, Vienna, Austria).

2 μm sections were stained with H&E (Carl-Roth, Vienna, Austria) according to the adapted manufacturer’s protocol. Sections were dewaxed and rehydrated as described above. After washing in water, H&E samples were incubated in solution 1 for 8 min, followed by 10 s rinsing in tap water and 10 s washing in 0.1% HCl. Samples were blued in running tap water for 4 min and stained with solution 2 for 45 s, followed by rinsing in tap water for 30 s.

Bright-field and fluorescence microscopy images were obtained using 10× magnification of an Olympus IX73 inverted microscope (Olympus Scientific Solutions, Vienna, Austria) using an excitation wavelength of 470 nm for lumogallion.

### 2.6 Aluminum in Air Pollution

Sample collection and measurements were performed alongside the air quality monitoring program of the Environment and Water Management Directorate of the State of Upper Austria, Austria. For detailed information, see Supporting Information Text S3. In short, particulate matter deposition was carried out in 18 different locations in Upper Austria, Austria, every fourth day throughout the whole year. At least one sample was measured per quarter, containing all fine dust continuously collected since the last measurement. Sample digestion was done according to DIN EN 14902. Measurements were performed using ICP–MS. The average recovery of Al was 60%, the LLOQ was 20 ng·m^–3^, and the LOD was 7 ng·m^–3^. Measurements were performed outside of the accredited laboratory area.

### 2.7 Statistical Analysis

Al concentrations of donors’ 1–8 tissues were tested for statistical significance using GraphPad Prism. Values were log-transformed and tested on normality (Shapiro–Wilk test). All data sets presented passed the test. Statistical comparison between selected tissues (dark gray bars) was done using one-way ANOVA with Tukey’s post hoc test. *p*-values <0.05 were considered statistically significant.

## 3 Results and Discussion

### 3.1 Patient Data

Patient data are shown in Table 1. The age ranged between 44 and 95 years. The type of residence was distinguished between city and country, especially indicating the kind of air pollution background exposure of individuals. Patient’s smoking status was evaluated at hospital admission before death, thus previous nicotine abuse cannot be ruled out. For information on the cause of death, pre-existing conditions, and medication, see Supporting Information Table S3. Selected tissues were weighed during the autopsies of patients 1–8, except for patient 2. Weights are shown in Supporting Information Table S4.

**Table 1:** Samples from 8 Male (m) and 12 Female (f) Patients Were Collected; NA = Not Available

patient	age (yrs)	sex (m/f)	height (cm)	weight (kg)	type of residence	smoking
1	75	F	151	52	country	no
2	64	m	180	95	country	yes
3	95	m	169	72	country	NA
4	56	m	185	95	country	NA
5	70	m	180	75	city	NA
6	59	f	160	64	city	NA
7	63	f	170	NA	city	no
8	73	f	160	100	city	no
9	85	f	NA	88	country	no
10	65	m	NA	66	city	yes
11	53	m	NA	NA	country	NA
12	74	f	164	86	country	NA
13	76	f	NA	102	city	NA
14	74	f	160	62	city	NA
15	92	f	NA	NA	city	NA
16	44	m	NA	NA	city	NA
17	92	m	NA	NA	city	NA
18	85	f	155	63	city	NA
19	80	f	NA	NA	country	NA
20	83	f	NA	NA	city	NA

### 3.2 ICP–MS Measurement of Al Concentration in Tissues Reveals High Concentrations in Lymph Nodes, Lungs, and Fingernails

Results of ICP–MS/MS measurements in μg of Al per kg of wet tissue are shown in Table 2. Any available instrumentation required for the drying of tissues is a potential source of Al contamination. Thus, wet weight was used for the measurement of Al concentrations in tissues, knowingly accepting a higher degree of uncertainty stemming from the use of wet weight but reducing the risk of contamination. Literature reports the lung, hilar lymph node, and heart water contents of 83.5 ± 2.1, 79.7 ± 2.0, and 78.3 ± 2.0% (average ± standard deviation), respectively.^33^

**Table 2:** Al Concentration of 24 Different Human Tissue Types (in μg/kg Wet Weight) Was Determined Using ICP–MS in a Clean Room^a^

Al (μg/kg)	patient 1	patient 2	patient 3	patient 4	patient 5	patient 6	patient 7	patient 8	median	SD
fingernail	14,000	22,400	6710	8600	65,900	5550^+^	4000	33,300	11,000	21,000
abdominal skin	962	290	721	306	755	2420	375	301	550	720
oral mucosa	512	<LOQ	321	<LOQ	<LOQ	6250	424	183	420	2600
cartilage	1450	401	<LOQ	218	124	2070	235	<LOQ	320	810
trachea	623	246	<LOQ	<LOQ	423	345	409	251	380	140
lung—right upper lobe	32,200	2120	20,700	3330	2190	1890	594	11,100	2800	12,000
lung—right inferior lobe	9060	1360	21,600	7430	831	954	746	1940	1700	7300
hilar lymph node	1,180,000	36,800	151,000	163,000	27,300	14,700	18,300	85,600	61,000	400,000
diaphragm	582	237	611	327	158	343	187	375	340	170
heart—left ventricle	10,400	524	<LOQ	174	164	112	241	194	190	3800
vena cava	605	550	377	191	136	345	497	403	390	170
thoracic aorta	701	269	304	182	129	500	156	330	290	190
intra-abdominal fat	227	219	182	<LOQ	<LOQ	317	105	138	200	75
stomach	537	293	311	325	129	374	223	4760	320	1600
duodenum	574	<LOQ	<LOQ	<LOQ	640	248	1160	240	570	380
ileum	543	288	<LOQ	195	474	406	283	292	290	120
colon	617	562	327	316	488	473	170	647	480	170
pancreas	<LOQ	<LOQ	250	<LOQ	153	254	100	<LOQ	200	76
kidney	206	<LOQ	<LOQ	<LOQ	136	233	139	130	140	47
spleen	691	<LOQ	468	487	242	117	<LOQ	143	360	230
liver	821	258	451	1130	236	216	249	174	250	350
urinary bladder	982	184	<LOQ	336	369	459	169	195	340	290
bone	19,400	1360	na	1050	2160	288	856	347	1100	7000
psoas major muscle	360	<LOQ	200	81.0	129	104	327	577	200	180

a No bone sample was provided for patient 3, and toenail instead of fingernail was provided for patient 6. <LOQ = below limit of quantification; ^+^ toenail instead of fingernail; NA = not available.

In Austria, skull openings during autopsies are performed only if indicated. Due to the rareness of these autopsies, the brain tissue was not considered for this study.

The highest concentrations were found in the hilar lymph node, fingernail, and upper and inferior lobes of the lung. With 1.18 g/kg, the hilar lymph node of patient 1 showed the highest concentration of all samples. For oral mucosa and bones, one sample showed an Al concentration at least ten times higher than the second highest concentration of the respective tissue type. Interestingly, both upper and inferior lobes of the lung showed very high Al concentrations, while tissues from the digestive system were among the samples with the lowest concentrations.

For the first time, the spotlight of human Al tissue distribution was on a large variety of tissue types, since studies so far have focused on the presumably most relevant tissues like brain, liver, and bone.^28^ Our broad approach using ICP–MS quantification under cleanroom conditions and thus a state-of-the-art setup surprisingly showed a high Al accumulation in lung tissues both in the upper and inferior lobes of the lung, as well as in hilar lymph nodes.^34−36^ This finding indicates that air might have been underestimated and underinvestigated as an Al source for the total human Al exposure until now. Previous literature has expressed the lack of inhalation data for Al.^28,37^ For example, one study investigating the accumulation of Al in 25 tissue types in mice did not include lungs.^38^ Notably, some studies have investigated various trace elements in lung tissues, some of which include Al.^33,39,40^ Discussion of the potential implications of Al found in lung tissues is missing in these publications, as this is beyond the scope of the respective work. Existing publications have estimated an Al absorption rate of 1.5–2% via the lung.^20,41^ It has previously been described that inhaled particles are partially deposited in hilar lymph nodes.^42,43^ We hypothesize that inhaled Al is also deposited in hilar lymph nodes.

**Figure 1:**
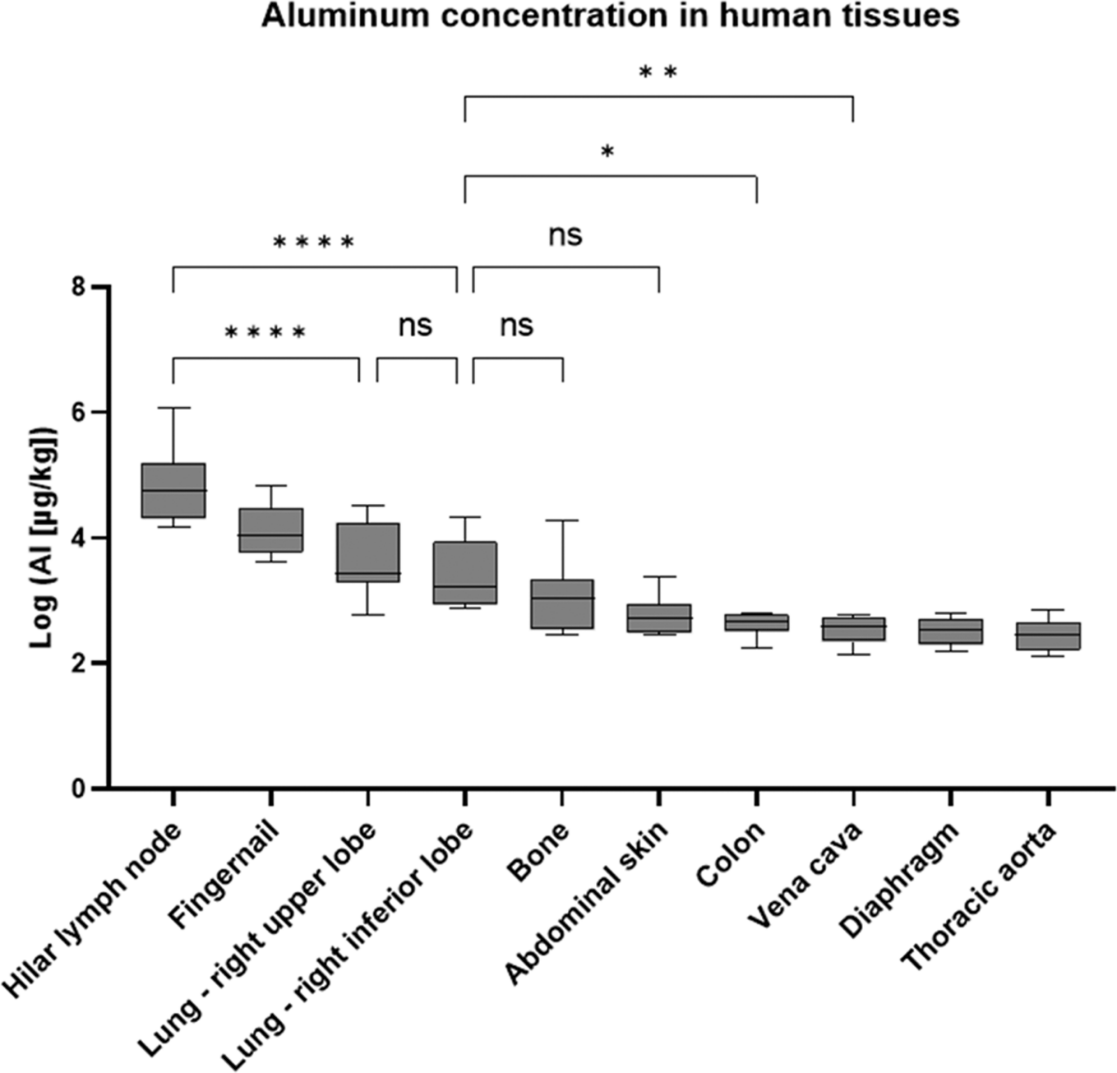
Statistically significant differences are shown for the following comparisons: hilar lymph node vs lung upper lobe; hilar lymph node vs lung inferior lobe; lung inferior lobe vs colon; lung inferior lobe vs vena cava. Data was log-transformed for better graphic representation. *p ≤ 0.05; **p ≤ 0.01; ****p ≤ 0.0001; ns not significant.

Statistical analysis of Al data for patients 1–8 was performed with all normally distributed tissue types, which include data from the five tissue types with the highest median Al concentrations (Figure 1). Not normally distributed data were mainly found for tissues with at least one value below LOQ. Hilar lymph nodes have significantly higher Al concentrations than both lobes of the lung (*p* ≤ 0.0001). No significant differences were found between the upper and inferior lobes of the lung (*p* > 0.05) and between the inferior lobe of the lung and bone tissue or abdominal skin, the two sample types with the next highest median Al concentration. Between the inferior lobe of the lung and colon (*p* ≤ 0.05) as well as vena cava (*p* ≤ 0.01), significant differences were found. For comparisons of all tissue types with normally distributed data, see Supporting Information Table S5.

Lungs are known to have high water content compared to other tissue types, thus the Al concentration measured was not the highest in tissue types with the lowest water content but seems to be independent of this factor.^44^ The water content of selected tissue types as described in the literature can be found in Supporting Information Table S6. The use of wet weight instead of dry weight is an important contribution to the total uncertainty of the presented results.

Previous literature shows the usability of toenail analysis for biomonitoring the history of exposure to various elements or determination of potential deficiencies of an element. The reported Al concentration in toenails was 26.91 μg/g, which is comparable to our measurements of fingernails, where a median concentration of 11 μg/g was found.^45^

### 3.3 8.7 mg of Total Al Was Found in the Average Lung

The absolute Al content of organs was calculated using the measured Al concentrations, and organ weights were determined during autopsies of patients 1–8. For calculations of the lung content, the average Al concentration of the upper and inferior lobes was used for each patient. No weights were provided for patient 2 and for the lung of patient 8.

The lowest average absolute Al amount was found in the kidney (0.03 mg), followed by the spleen (0.04 mg) and heart (0.45 mg). For detailed information, see Supporting Information Table S7. The liver content was found to be 0.81 mg, which is only one tenth of the average lung content of 8.7 mg. Overall, the lung of patient 1 had a total of 19.5 mg of Al, while the lung of patient 7 only had 0.6 mg. This might be due to individual Al exposure throughout life.

### 3.4 Al/Si Ratio in Lungs and Lymph Nodes Is Comparable to the Al/Si Ratio in PM_10_

Al and Si levels in urban PM_10_ are known to correlate, as similar sources of these elements are expected, especially geogenic sources and resuspended road dust.^46^ Al/Si ratios previously reported in PM_10_ are 0.27,^46^ 0.28,^47^ 0.20, and 0.47.^48^ We found an average Al/Si ratio (mean ± standard deviation) of 0.56 ± 0.15 (upper lobe of the lung), 0.51 ± 0.14 (inferior lobe of the lung), and 0.49 ± 0.12 (hilar lymph node; see Table 3). Interestingly, individual concentrations vary greatly, but patients with a high Al content also show a high Si content, and vice versa. Given that the matrixes of human tissues and PM_10_ are indeed quite different, and thus the relevant sample preparation methods, we considered the Al/Si ratio measured in lung and hilar lymph node tissues comparable to that found in urban PM_10_.

**Table 3:** Al and Si Concentrations Found in the Hilar Lymph Node and Upper and Inferior Lobes of the Lung of Patients 9–20, as Well as the Average Al/Si Ratio for the Respective Tissues

	hilar lymph node			lung—right upper lobe			lung—right inferior lobe		
patient	Al (mg/kg)	Si (mg/kg)	Al/Si	Al (mg/kg)	Si (mg/kg)	Al/Si	Al (mg/kg)	Si (mg/kg)	Al/Si
9	4.61	13.7	0.34	5.87	11.4	0.52	3.19	9.50	0.34
10	223	482	0.46	6.99	16.5	0.42	11.6	21.5	0.54
11	32.2	48.1	0.67	14.5	30.1	0.48	27.9	54.0	0.52
12	119	255	0.47	9.28	21.1	0.44	8.92	15.6	0.57
13	193	325	0.60	2.19	3.61	0.61	2.74	7.86	0.35
14	22.3	41.2	0.54	2.25	6.46	0.35	1.12	2.79	0.40
15	69.8	174	0.40	41.6	63.2	0.66	18.2	37.4	0.49
16	7.30	20.1	0.36	4.52	9.88	0.46	7.08	11.0	0.64
17	300	499	0.60	22.0	36.6	0.60	30.5	48.8	0.63
18	87.9	169	0.52	2.95	3.74	0.79	5.47	7.69	0.71
19	13.8	47.7	0.29	9.77	11.4	0.85	1.68	5.81	0.29
20	119	200	0.59	4.98	10.0	0.50	5.37	9.06	0.59
average	99.3	190	0.49	10.6	18.7	0.56	10.3	19.3	0.51
SD	95.9	172	0.12	11.3	17.3	0.15	10.0	17.6	0.14

This strongly indicates that fine dust is, indeed, a relevant human Al source. A possible explanation for the slightly higher Al/Si ratio found in human lung and hilar lymph node tissues compared to that in urban fine dust is the presence of additional nonairborne sources of human Al.

### 3.5 Histological Staining Confirms the Uptake of Al in Lungs

**Figure 2:**
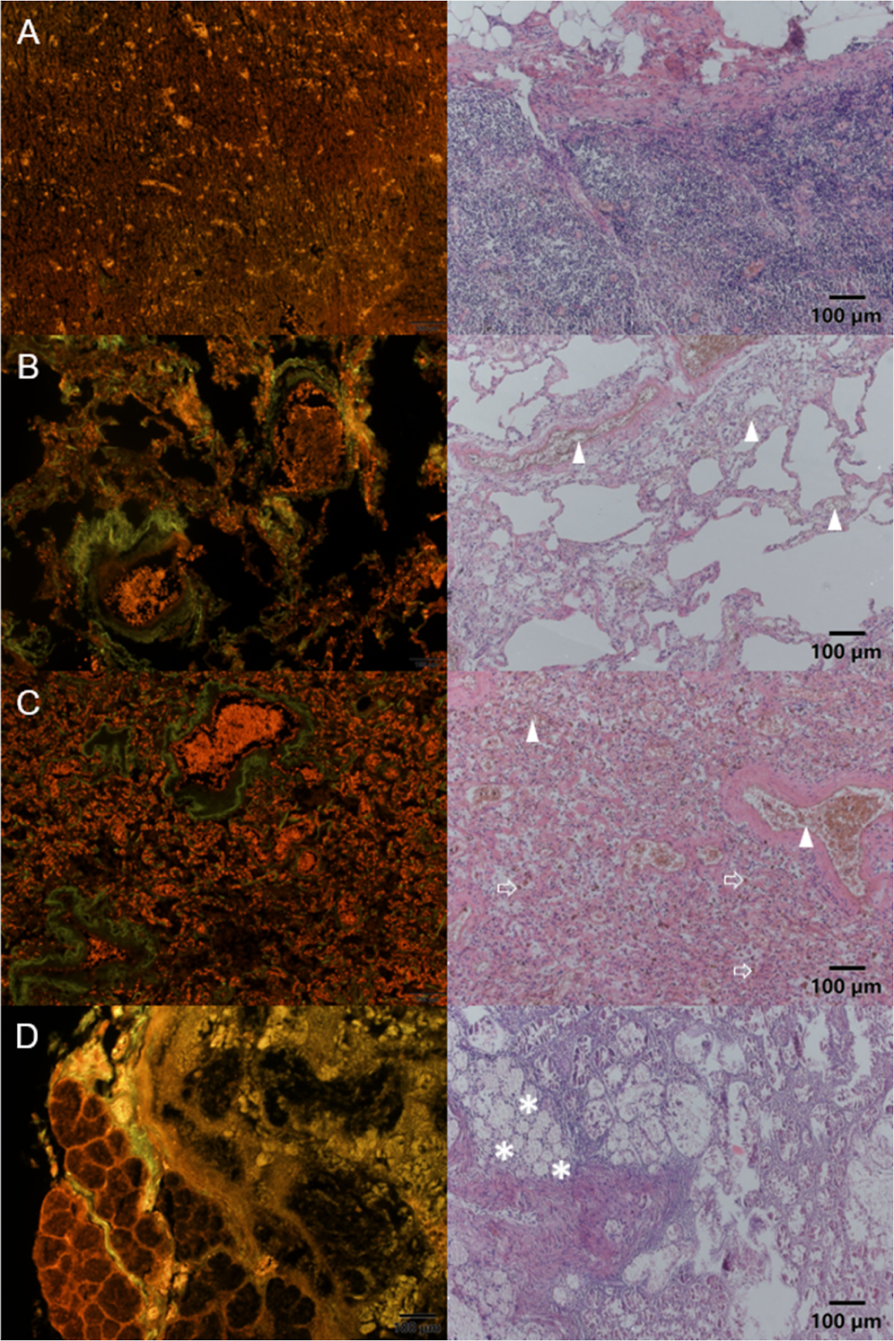
Lumogallion (left column, red Al-specific fluorescence) and H&E images (right column) of the hilar lymph node (A), upper lobe of the lung (B), inferior lobe of the lung (C), and duodenum (D) are shown for patient 4. Erythrocytes are stained red using H&E (indicated with triangles) and can be found inside blood vessels as well as within the tissue of upper and inferior lobes of the lung. The location of erythrocytes in H&E and that of Al signals in lumogallion staining overlap, showing that Al is bound in erythrocytes in lung tissues. Macrophages are prominent in C (larger dark-red areas outside blood vessels, indicated with arrows) and also contain Al. Brunner’s glands showed high Al signals and could be identified on H&E-stained slides (indicated with asterisks).

Formalin-fixed paraffin-embedded (FFPE) slides were prepared for all tissue types except for bones and fingernails. The upper and inferior lobes of the lung, hilar lymph nodes, and the duodenum were stained with lumogallion and H&E (see Figures 2 and S1). Lungs and lymph nodes were chosen due to their high Al concentrations, while the duodenum was used as an example of a tissue type with a low Al content. Green to yellow autofluorescence of most tissue types and Al-specific red-orange fluorescence which appears as bright, small spots (emission maximum: 580 nm) were observed. Since all detected Al accumulation in specific regions of tissue samples was found in all patient samples of the same tissue type and relative concentrations between different tissue types were consistent with concentrations measured with ICP–MS, contamination as a cause of all Al signals could be ruled out.

We could clearly show that Al is abundantly present in both upper and inferior lobes of the lung as well as lymph nodes, while it was only present in specific regions of duodenum (see Figures 2 and S1). In lungs, Al is found in erythrocytes, among other regions, indicating the transfer of Al taken up from air into red blood cells and thus into the bloodstream (Figure 2B,C). Patient 4 showed an edema in the inferior lobe of the lung, thus more alveolar macrophages were present in the tissue. Alveolar macrophages are responsible for the transport of unusable particles into hilar lymph nodes and are also found to contain Al (see Figure 2C). In the hilar lymph node, Al can be found in capillaries (see Figure 2A). In the duodenum, almost exclusively Brunner’s glands were found to contain Al (see Figure 2D).

Previous studies have shown that airborne fine particles are taken up via lungs, reach the blood circulation, and translocate to different organs like the brain, heart, and placenta.^49−51^ PM can cross the lung–blood barrier through two pathways: by itself, which depends on factors like size, charge, and chemical composition of particles, and via ingestion of alveolar macrophages.^52^ Inhaled particles accumulate in hilar lymph nodes, where concentrations are 1–20-fold higher than those in lung tissues.^53^

We argue that Al is transported into erythrocytes in the lungs rather than it being taken up by a different organ and transported to the lung via red blood cells because in the latter case, other organs with a high blood flow or blood vessels would be expected to show similarly high Al concentrations as the lung, which is clearly not the case. Notably, our experiments cannot prove that Al observed in lung erythrocytes originates from airborne particles, which needs to be investigated in future work.

Previous studies have used lumogallion staining and fluorescence microscopy for the detection and visualization of Al within paraffin-embedded tissues and viable cells.^32,54−56^ This relative quantitative method can help to understand the distribution of Al within samples, but it cannot absolutely quantify Al or replace ICP–MS/MS quantification. Limitations of this staining method are the fact that lumogallion only binds to free Al^3+^ but not to complexed Al, as it would be present in food color lake pigments. Thus, Al originating from complexes such as, for example, lake food colors, cannot be visualized with lumogallion. As coordination complexes are not expected to be of high relevance for airborne Al, we accepted this limitation of the lumogallion. Furthermore, working outside a cleanroom, contamination with Al cannot be ruled out. In this study, lumogallion staining was used to understand the distribution of Al within the tissue types. For the investigated tissues, the same distribution pattern was found across all 6 patients for which FFPE samples were available, showing the adequacy of this technique. Despite the high Al concentrations found in fingernails, these samples were not further investigated with lumogallion because of the small size of provided samples and the uniform structure of fingernails.

Surprisingly, we could show that Al is not solely deposited in lung tissues but that Al is present in erythrocytes and macrophages in lung alveoli. This indicates that airborne Al is taken up into the bloodstream, where it can be distributed within the body. Understanding the underlying mechanism of uptake and transport of airborne Al requires further investigations.

### 3.6 Aluminum in Air Pollution

Because of the high Al concentrations found in the lungs across all patients, Al concentrations of PM_10_ and PM_2.5_ were investigated. The aim of this study was not to absolutely quantify Al in particulate matter but to find out if it can be detected at all. There is a lack of data for Al in air pollution, and no official monitoring of airborne Al is required by law, for example, through the European Union.

Al was detected across all 18 sampling locations in Upper Austria and in every year of sampling. The highest average of 151 ng/m^3^ was found in quarter 2 (Q2) of Enns/Kristein, while Q4 of Berufsschule Wels showed the lowest average with 67 ng/m^3^. Quarterly averages of five locations where data were available for 2015–2020 (Berufsschule Wels) or 2014–2020 (Römerberg Linz, Enns/Kristein, Stadtpark Linz and Neue Welt Linz) are shown in Supporting Information Figure S2. Data were kindly provided by the Environment and Water Management Directorate of the state of Upper Austria, Austria.

Even though the recovery of Al for the measurements is not ideal, it does not exceed 100% and behaves similarly across the span of a year at all locations and throughout the entire measurement period (see Supporting Information Figure S2), which indicates no substantial contamination and the potential of the setup to detect Al. The sample collection and measurement were not done specifically to detect Al but was performed alongside the air quality monitoring program of the State of Upper Austria, Austria. Thus, the procedure was not optimized for Al quantification, and a recovery of about 100% was not expected. We much rather aimed for reliably and repeatedly detecting Al in PM. Thus, the finding of Al in all samples taken showed that there is, in fact, Al present in PM, which might explain the high concentrations found in lungs and lymph nodes.

In conclusion, ICP–MS measurements of 24 different tissue types in 8 patients surprisingly showed that hilar lymph nodes and upper and inferior lobes of the lung exhibit the highest Al concentrations in humans (61,000, 2800, and 1700 μg/kg, respectively), which has not been described previously. Up to 8.7 mg of total Al was found in the lung tissue, where Al was found to be present mainly in erythrocytes and macrophages. The Al/Si ratios found in lung and hilar lymph node tissues of additional 12 patients are comparable to that reported in PM_10_ in the literature. Together with Al constantly found in PM_10_ and PM_2.5_ measurements in different geographical locations in Upper Austria over 7 consecutive years, we could clearly show that the pulmonary content of Al is substantial and has previously been underestimated. Further investigations of airborne Al, its potential pulmonary uptake, and implications for both humans and the environment are strongly indicated to rule out potential harmful effects of this so far underestimated Al source.

## Supporting Information Available

Chemicals and reagents, sample preparation, and ICP–MS/MS measurements of patients 1–8; ICP–MS parameters for tissue Al measurements; chemicals and reagents, sample preparation, and ICP–SFMS measurements of patients 9–20; ICP–SFMS parameters; patient information; organ weights; statistical information; tissue water content; total Al tissue content; lumogallion and H&E staining of patient 2; and quarterly averages of Al in PM10 between 2014 and 2020 (PDF)
