# Supplementary material for: Airborne Aluminum as an Underestimated Source of Human Exposure: Quantification of Aluminum in 24 Human Tissue Types Reveals High Aluminum Concentrations in Lung and Hilar Lymph Node Tissues
Source: Environ Sci Technol. 2024 Jun 18;58(26):11292–300. doi: 10.1021/acs.est.4c01910 (PMC11223461; doi:10.1021/acs.est.4c01910)
Supplement: Supplementary file 1 — es4c01910_si_001.pdf [file es4c01910_si_001.pdf]

Supporting Information for

Airborne Aluminum as an Underestimated Source of Human Exposure: Quantification of Aluminum in 24 Human Tissue Types Reveals High Aluminum Concentrations in Lung and Hilar Lymph Node Tissues

Clara Ganhör[a], Lukas Mayr[a], Julia Zolles[b], Marion Almeder[c], Matin Kazemi[a], Markus Mandl[a], Christian Wechselberger[a], Dave Bandke[c], Sarah Theiner[b], Christian Doppler[a*], Andreas Schweikert[b], Marina Müller[a], Špela Puh[a], Michaela Kotnik[a], Rupert Langer[c], Gunda Koellensperger[b], David Bernhard[a, d]

[a] Division of Pathophysiology, Institute of Physiology and Pathophysiology, Medical Faculty, Johannes Kepler University, Linz, Austria

[b] Institute of Analytical Chemistry, Faculty of Chemistry, University of Vienna, Vienna, Austria

[c] Institute of Clinical Pathology and Molecular Pathology, Kepler University Hospital and Johannes Kepler University, Linz, Austria

[d] Clinical Research Institute for Cardiovascular and Metabolic Diseases, Medical Faculty, Johannes Kepler University, Linz, Austria

* *Corresponding author at:* christian.doppler@jku.at

Number of Pages: 20

Number of Figures: 2

Number of Tables: 7

Table of Contents

Text S1. Chemicals and reagents, sample preparation and ICP-MS/MS measurements of patients 1-8 3

Table S1. ICP-MS parameters for tissue Al measurements 6

Text S2. Chemicals and reagents, sample preparation and ICP-SFMS measurement of patients 9-20 7

Table S2. ICP-SFMS parameters 9

Table S3. Patient information 12

Table S4. Organ weights 15

Table S5. Statistical information 16

Table S6. Tissue water content 17

Table S7. Total Al tissue content 18

Figure S1. Lumogallion and H&E staining of patient 2 19

Figure S2. Quarterly averages of Al in PM10 between 2014-2020 20

Supplementary References 21

Text S1. Chemicals and reagents, sample preparation and ICP-MS/MS measurements of patients 1-8

Samples of patients 1-8 were divided into three sets, every set was measured on a different day. No bone sample was provided for patient 3, and toenail instead of fingernail was provided for patient 6.

Chemicals and reagents

Ultrapure water (18.2 MΩ cm) was purified using the ELGA Water Purification System (Purelab Ultra MK 2, United Kingdom). HNO_3 Suprapur® (*w* = 65%) was purchased from Supelco® (VWR, Vienna, Austria). H_2O_2 Suprapur® (*w* = 30%) was purchased from Merck (Vienna, Austria). All utensils were cleaned in 10% HNO_3 Suprapur® for 24 hours, followed by 24 hours in 1% HNO_3 Suprapur® and washing with ultrapure water.

To validate trueness, the certified reference material BCR-639 (Joint Research Centre, Institute for Reference Materials and Measurements, European Commission, Geel, Belgium) was used for reference extractions. Furthermore, In and Re were used as internal standards during IP-MS/MS measurements. Both were used as single element standards with a concentration of 1000 +/- 3 mg · L^{-1-} and were purchased from LabKings (Hilversum, the Netherlands). The quality control 26-element standard solution for calibration was purchased from LabKings (Hilversum, the Netherlands).

Sample preparation was performed in a cleanroom of ISO class 8, ICP-MS/MS measurements were performed in an ISO class 7 clean room.

Sample preparation for elemental analysis

After delivery to the laboratory, 5 mL of 30% HNO_3 were added to each frozen (-18 °C) sample and transferred to PFA extraction tubes. Two BCR-639 plasma reference material and four blank extractions only containing HNO_3 were included in every extraction run. During the first three extraction days, 250 µL of reference material were weighed and used for reference material extraction. Measurements were close to or below LLOQ, thus the volume was increased to 500 µL for all following reference material

extractions. In total, 16 extractions with 500 µL, which were weighed before digestion, were performed yielding a median Al concentration of 214 +/- 12 µg/L (median +/- standard deviation), which is in the certified range of 194 +/- 14 µg/L. Due to high instrumentation cleaning efforts required after ICP-MS measurement of patients 1-5, 1 mL of 30% H_2O_2 was added to all extraction tubes of patients 6-8 to ensure full digestion of samples. Anton Paar Multiwave PRO was used for microwave digestion, where temperature increased to 180°C within 20 minutes, was constant at 180°C for 10 minutes followed by cooling to 70°C. Extracts were transferred into metal free tubes, diluted with 5 mL ultrapure water or, for samples containing 1 mL H_2O_2, 4 mL ultrapure water, and weighed. Samples were diluted 1 to 5 with ultrapure water by weighing, to reach a total HNO_3 concentration of approximately 3%. Extracts with Al concentrations exceeding 1000 ppb were diluted 1 to 10 with 3% HNO_3. After each sample digestion cycle, one cleaning cycle using HNO_3 and applying the microwave program as for the sample digestion was performed to minimize the risk for carryover.

Quantification was performed by a 9-point matrix-matched external calibration with standard solutions containing Al ranging from 0.1 $\mu g \cdot L^{-1}$ – 200 $\mu g \cdot L^{-1}$. To validate trueness, certified reference material TM 35.2 (trace element matrix reference material made from filtered and diluted Lake Ontario water, certified for Al) was measured with every sample set.

The plasma reference material BCR-639 provides certified values for Al, Se and Zn. The sample preparation procedure and ICP-MS analysis were also suitable for the analysis of Se and Zn, which were thus also quantified. The clear focus of this study is Al, therefore the results of Se and Zn measurements were not provided in this publication.

ICP-MS/MS measurements

Inductively coupled plasma mass spectrometry (ICP-MS/MS) measurements were performed using Agilent 8800 ICP-MS/MS (Agilent Technologies, Tokyo, Japan) equipped with an CETAC ASX-520 autosampler (CETAC Technologies, Omaha, USA) and a MicroMist nebulizer operated at a sample

uptake rate of approximately 0.25 mL/min. For ICP-MS/MS calibration, Quality Control Standard #26 (LabKings, Hilversum, The Netherlands) was used. The Agilent MassHunter software packages (Workstation Software, Version C.01.03, 2019) was used for data evaluation. The instrumental parameters are summarized in Supporting Table S1.

Table S1. ICP-MS parameters for tissue Al measurements

Table S1. *Instrumental parameters for ICP-MS/MS measurements of tissue Al concentrations*

ICP-MS/MS parameters	
RF power	1550 W
Sampling depth	8.0 mm
Nebulizer	MicroMist
Spray chamber	Scott double-pass
Spraying chamber temp.	2°C
Monitored Isotopes	$^{27}Al \rightarrow ^{43}Al$, ^{64}Zn, ^{82}Se, $^{82}Se \rightarrow ^{98}Se$, ^{115}In, ^{185}Re
Measurement modes	no gas, O_2,
Plasma gas	15 L/min
Nebulizer gas	1.00 L/min
Auxiliary gas	0.90 L/min
Cones	Ni
Cell entrance	-40 V
Cell exit	-50 V
Integration time	0.1 - 0.5 s

Text S2. Chemicals and reagents, sample preparation and ICP-SFMS measurement of patients 9-20

Chemicals and reagents

Laboratory water type I (Milli-Q® water, 18.2 MΩ · cm, SG Water GmbH, Barsbüttel, Germany) was purified using a duoPUR Quartz sub-boiling-system (Milestone-MLS GmbH, Leutkirch, Germany). Pro analysis (p.a.) grade HNO_3 (w = 65 %) was obtained from Merck (Darmstadt, Germany). H_2O_2 suprapur® (w = 30 %), HF suprapur® (w = 40 %) and H_3BO_3 suprapur® were obtained from Supelco®. HNO_3 was sub-boiled using a Savillex DST-1000 acid purification-system. Certified ICP single element standards for trace analysis were purchased from Inorganic Ventures (Al, Si and In at concentration of 1000 mg L^{-1}). All laboratory consumables used for sample preparation and analysis were made of plastic material and double acid cleaned (HNO_3/HCl, w = 10 %/2 % (v/v) and w = 1 %/0.2 % (v/v) 24 h each, rinsed three times with Milli-Q®-water and dried in a clean bench before single use. 50 mL PE bottles used for sample storage were conditioned with 10 % (v/v) sub-boiled HNO_3/0.2 % (v/v) HF after the acid cleaning procedure until further use.

To validate trueness, certified reference materials TM 35 (trace element matrix reference material made from filtered and diluted Lake Ontario water, certified for Al) and BOVM-1 Bovine Muscle Certified Reference Material for Trace Metals and other Constituents (National Research Council Canada, information value for Al given), have been processed. Additionally, spike recovery experiments at two different concentration levels were conducted.

Preparatory laboratory work for elemental analysis and measurements were performed in an ISO class 8 and an ISO class 7 clean room with laminar flow hoods ISO class 5 according to ISO 14644-1, respectively.

Sample preparation for elemental analysis

After delivery to the laboratory, the frozen (-18 °C) tissue samples were mineralized by microwave assisted acid digestion. Approximately 0.300 g of sample were accurately weighted into PTFE vessels

of the microwave (Anton Paar, Graz, Austria, Multiwave 3000, rotor 16 HF 100). In a first step, sample digestion was performed with 4 mL of concentrated sub-boiled HNO_3, 1 mL of H_2O_2 and 50 µL of HF at 1400 W for 15 min (+ 15 min ramp). In a second step, complexation of HF with H_3BO_3 was performed. Therefore, the microwave vessels were quickly opened after cooling down to approx. 30 °C, 500 µL of super-saturated re-crystallized H_3BO_3 were added, vessels were closed again and another digestion cycle was performed at 1000 W for 10 min (+ 15 min ramp). After cooling down the vessels to room temperature, the obtained digests were quantitatively transferred into 50 mL mL PP-bottles and gravimetrically diluted to 30 g with sub-boiled water. After each sample digestion cycle, two cleaning cycles, using HNO_3/H_2O_2/HF and applying the microwave program as for the sample digestions were performed to minimize the risk of analyte carry-over.

For analysis, the samples were, depending on the concentrations of the analytes, not diluted and further gravimetrically diluted by a factor of 5, 50 and 100, respectively, to fit within the applied working range (0.1 – 100 µg L^{-1}) and spiked with the internal standard to obtain a final concentration of 1 µg L^{-1} In in the sample. To minimize matrix induced measurement- and quantification errors, dilutions were performed using diluted digestion blanks and keeping the acid concentrations constant.

Quantification was performed by an 8-point matrix-matched external calibration with standard solutions of Al and Si ranging from 0.1 µg L^{-1} – 100 µg L^{-1} and internal standardization via In. The CRM TM 35 was used for verification of the analytical method. To verify matrix induced signal intensities and resulting slopes, calibration standards were prepared in synthetic solutions containing 13.3 % (v/m) HNO_3 + 0.17 % (v/m) HF, and solutions obtained by dilutions of digestion blanks and digested BOVM-1 with 13.3 % (v/m) HNO_3 + 0.17 % (v/m) HF, respectively, by a factor of 10 and 100.

ICP-SFMS measurements

Quantification was performed on the ICP-SFMS *Element2* High Resolution ICP-SFMS (ThermoFisher, Bremen, Germany) in the high-resolution mode (HR, $m/\Delta m$ >10000), equipped with a PFA scott-type

spray chamber and an injector made of sapphire (both AHF Analysentechnik). Measurements were performed by continuous acquisition (sample intake via PFA-ST nebulizer and a 0.2 mm i.d. uptake capillary (Elemental Scientific Inc,. ESI, Omaha) in self-aspiration mode (sample uptake:50 µL min^{-1}). Tuning parameters were optimized to obtain best possible sensitivity (min $1x10^6$ cps/1 µg L^{-1} In in LR), signal stability and an oxide rate $^{238}U^{16}O^{+}/^{238}U^{+}$ ratio <5%. Instrumental parameters and masses monitored are shown in Table S2.

Table S2. ICP-SFMS parameters

Table S2. *Instrumental parameters for ICP-SFMS measurements of Al and Si in tissues of patients 9-20.*

Parameter	Value
Plasma [W]	1200
Plasma gas flow [L min^{-1}]	16
Auxiliary gas flow [L min^{-1}]	0.9
Nebulizer gas flow [L min^{-1}]/	1.186
Additional gas flow [L min^{-1}]	0.1
HR mode	^{27}Al,' ^{28}Si, ^{29}Si and ^{30}Si, ^{115}In (internal standard)
Data acquisition	E-scan mode, 3 runs*3 passes, 80% mass window, 45% integration window, 100 samples/peak, 800 ms

Text S3. Al measurement in PM_{10} and $PM_{2.5}$

Sample collection

High volume aerosol sampler DHA-80 with an inlet for PM_{10} and $PM_{2.5}$ sampling (DIGITEL enviro-sense, Bürs, Austria) was used for airborne particle collection on quartz fiber filters. Samples were taken by the Department of Environmental Protection of the Environment and Water Management Directorate in the state of Upper Austria, Austria, for the governmental air quality monitoring program in accordance with the EU Ambient Air Quality Directive.

Sample preparation and analysis

Sample preparation was performed using nitric acid, hydrogen peroxide and hydrochloric acid according to DIN EN 14902. Reference material ERM-CZ120 (Joint Research Centre, Institute for Reference Materials and Measurements, European Commission, Geel, Belgium) was used to calculate the recovery of Al. ICP-MS measurements were performed using NexION 300D ICP-MS (PerkinElmer, Rodgau, Germany).

Data analysis

In total, data was provided for 18 locations. Continuous quarterly data for 2014-2020 was available for four locations (Enns/Kristein, Neue Welt, Römerberg, Stadtpark). In the case of the location Berufsschule Wels, continuous quarterly data was available for 2015-2020. Al data of these five locations was analyzed. Overall, data for three quarters was missing for these five locations over the entire sampling period. Al concentrations in PM_{10} were provided for all five sampling sites. Furthermore, for Berufsschule Wels and Stadtpark, data for $PM_{2.5}$ was also provided. Quarterly averages of PM_{10} for the five locations available and the average of these sites are given in Figure S2. Al was detected across all locations and all years, with the highest average of 151 $ng \cdot m^{-3}$ found in Q2 of Enns/Kristein (data from 2014-2020), while Q4 of Berufsschule Wels showed the lowest average with

67 ng · m^{-3} (data from 2015-2020). $PM_{2.5}$ values were always lower than PM_{10} values, which is in accordance with expectations because $PM_{2.5}$ particles are included in PM_{10}.

Table S3. Patient information

Table S3. Information on cause of death, pre-existing conditions and medications of patients 1-20.

Patient	Cause of death	Pre-existing conditions	Medication
1	acute necrotizing pancreatitis with peritonitis	septic shock, hypothyreosis, acute renal failure, pancreatitis, arterial hypertension, colon carcinoma, hypercholesterolemia, appendectomy	Euthyrox, Lisinopril, Simvastatin
2	respiratory arrest / apoplex	coronary heart disease, hypercholesterolemia, prediabetes, chronic nicotine abuse, arterial hypertension	Candecam, Concor, Atorvastatin, Thrombo ASS, Nitro spray
3	fat embolism	coronary heart disease, status post stenting, spinal canal stenosis, dementia, status post cysticus stent, status post embolectomy	Hidrasec, Lixiana, Lovenox, Novalgin, Paracetamol, Piritramid, Bicalutamid, Candeblo, Laevolac, Laxis
4	massive aspiration	asystolia	NA
5	cardiorespiratory failure	structural epilepsy, dementia onset, arterial hypertension, suspected stroke, non-small cell liver carcinoma	Amlodipine, Atorvastatin, Combivent, Ebrantil, Isosource Standard Fibre, Lasix, Lovenox, Molaxole, Thrombo ASS, Zyvoxid, Oxygen
6	cardiac decompensation	coronary heart disease, mitral valve insufficiency, aortic aneurysm, hypertension	Ramipril, Bisoprolol, Rosuvastatin, Thrombo ASS, Lasix
7	liver failure	hypermenorrhea, uterus myomatosus, suspected head of pancreas carcinoma, painless icterus, non-small cell carcinoma	Piperacillin, Elomel, Pantoprazol, Lovenox, Fortimel, Metamizol, Zofran
8	liver failure	lymphadenopathy, arterial hypertension, renal colic, suspected metastasizing cervic carcinoma	NA
9	heart failure	endometrium carcinoma, adipositas, diabetes, arterial hypertension, chronic renal insufficiency, cardiac decompensation	Herion, Novomix, Paracodin, Vendal
10	cardiac insufficiency with renal failure	diabetes, chronic obstructive pulmonary disease, arterial hypertension, chronic renal insufficiency, dementia, cardiac	Atorvastatin, Berodual Spray, Burinex Leo, Combivent,

		decompensation, pleural effusion	Doxazosin, Lasix, Nephrotrans, Novorapid, Prednisolut
11	cardiorespiratory failure with pulmonary embolism	rupture of aortic wall, arterial hypertension, hypercholesterolemia, diabetes, frozen elephant trunk	Amlodipin, Calciduran, Euthyrox, Ferretab, Metafelan, Metohexal, Pantip, Paracetamol, Ramicomp, Rosovastatin, Thrombo ASS, Synjardy
12	peritonitis	adipositas, arterial hypertension, endometrium carcinoma, arterial fibrillation	Dormicum, Novalgin, Cefotaxim, Diclobene, Elomel, Folsan, Xarelto, Lovenox, Metropolol, Metronidazole, Paracetamol, Piritramid, Ramipril, Rosovastatin
13	onset of pneumonia with cardiac hypertrophy	hypercholesterolemia, diabetes, arterial hypertension, coronary heart disease, fatty liver	ASS Hexal, Ezegelan, Gliclazid, Rosuvastatin
14	respiratory insufficiency	trigeminal neuralgia, polyneuropathy, status epilepticus, depression, irritable colon, suspected pneumonia	Airvo Fio2, Combivent, Elozell Soezial, Fycompa, Lacosamid, Lasix, LEvebon, Lovenox, Vendal, Psychopax, Quetialan, Unasyn
15	cardiorespiratory failure	pneumonia, hyponatremia, urinary tract infection	Lovenox, Metegelan, Paracetamol, Fenistil, Halcion, Metamizol
16	progressive tumor with metastases	urothelial carcinoma with metastases	Calciduran, Calcitonin, Dronabinol, Fragmin, Haldol, Hydal, Ibuprofen, Lasix, Metamizol, Mirtabene, Molaxole, Piperacil, Pregabalin, urosin, Voltaren, Vendal, Zofran, Zoldem, Zyvoxid
17	respiratory	arterial hypertension, ischemic	Ceftriaxon, Concor,

	insufficiency	cardiomyopathy, dyspnoea, covid-19	Dutasterid, Eliquis, Molaxole, Patnip, Rosovastatin, Sifrol, Teveten, Tritico, Vendal
18	cardiorespiratory insufficiency in pneumonia and inner layer infarction	hypertension, mitral insufficiency, interstitial lung disease, atrial fibrillation, basalioma	Amlodipin, Combivent, Eliquis, Latanovision, Pantoprazol, Prednisolon, Zithromax, Fenistil, Fentanyl, Lasix, Metagelan, Piperacillin, Vendal
19	decompensated heart failure	chronic obstructive pulmonary disease, coronary heart disease, Alzheimer's disease, permanent atrial fibrillation	Combivent, Lovenox, Novalgin, PCA, Piperacillin, Mirtabene, Molaxole, Pantolol Sertralin, Euthyrox
20	cardiac decompensation	chronic renal insufficiency, ischemic cardiomyopathy, covid-19, gout arthropathy	Ceolat, Dicloakut, Dilatrend, Dominal, Enterobene, Entresto, Ezegelan, Unasyn, Lasix, Lecicarbon, Lexotanil, Metegelan, Microlax, Prednisolon, Rosuvastatin, Temesta

Table S4. Organ weights

Table S4. For all patients except patient 2, organ weights of heart, lungs, liver, spleen and kidneys were determined during autopsy. NA = not available

Patient	Heart (g)	Lungs (g)	Liver (g)	Spleen (g)	Kidneys left+right (g)
1	265	945	970	100	215
2	NA	NA	NA	NA	NA
3	480	960	1,238	124	235
4	460	1,475	1,860	230	300
5	355	1,565	1,320	85	275
6	720	1,010	1,260	120	255
7	400	875	4,470	285	310
8	450	NA	3,105	250	385

Table S5. Statistical information

Table S5. Data was tested for statistical significance between all of the following tissue types: fingernail, abdominal skin, lung - right upper lobe, lung - right inferior lobe, hilar lymph node, diaphragm, vena cava, thoracic aorta, bone; These tissues showed normally distributed data. * $p \leq 0.05$; ** $p \leq 0.01$; *** ≤ 0.001; **** $p \leq 0.0001$; *ns not significant*

Tukey's multiple comparisons test	Summary	Adjusted P Value
Fingernail vs. Abdominal skin	****	<0.0001
Fingernail vs. Lung - right upper lobe	ns	0.4613
Fingernail vs. Lung - right inferior lobe	ns	0.0574
Fingernail vs. Hilar lymph node	*	0.0365
Fingernail vs. Diaphragm	****	<0.0001
Fingernail vs. Vena cava	****	<0.0001
Fingernail vs. Thoracic aorta	****	<0.0001
Fingernail vs. Colon	****	<0.0001
Fingernail vs. Bone	***	0.001
Abdominal skin vs. Lung - right upper lobe	**	0.0052
Abdominal skin vs. Lung - right inferior lobe	ns	0.0938
Abdominal skin vs. Hilar lymph node	****	<0.0001
Abdominal skin vs. Diaphragm	ns	0.9701
Abdominal skin vs. Vena cava	ns	0.9908
Abdominal skin vs. Thoracic aorta	ns	0.897
Abdominal skin vs. Colon	ns	0.9997
Abdominal skin vs. Bone	ns	0.8856
Lung - right upper lobe vs. Lung - right inferior lobe	ns	0.9908
Lung - right upper lobe vs. Hilar lymph node	****	<0.0001
Lung - right upper lobe vs. Diaphragm	****	<0.0001
Lung - right upper lobe vs. Vena cava	***	0.0001
Lung - right upper lobe vs. Thoracic aorta	****	<0.0001
Lung - right upper lobe vs. Colon	***	0.0005

Lung - right upper lobe vs. Bone	ns	0.3479
Lung - right inferior lobe vs. Hilar lymph node	****	<0.0001
Lung - right inferior lobe vs. Diaphragm	**	0.0027
Lung - right inferior lobe vs. Vena cava	**	0.0052
Lung - right inferior lobe vs. Thoracic aorta	**	0.0011
Lung - right inferior lobe vs. Colon	*	0.0155
Lung - right inferior lobe vs. Bone	ns	0.9183
Hilar lymph node vs. Diaphragm	****	<0.0001
Hilar lymph node vs. Vena cava	****	<0.0001
Hilar lymph node vs. Thoracic aorta	****	<0.0001
Hilar lymph node vs. Colon	****	<0.0001
Hilar lymph node vs. Bone	****	<0.0001
Diaphragm vs. Vena cava	ns	>0.9999
Diaphragm vs. Thoracic aorta	ns	>0.9999
Diaphragm vs. Colon	ns	>0.9999
Diaphragm vs. Bone	ns	0.2093
Vena cava vs. Thoracic aorta	ns	>0.9999
Vena cava vs. Colon	ns	>0.9999
Vena cava vs. Bone	ns	0.2981
Thoracic aorta vs. Colon	ns	0.9981
Thoracic aorta vs. Bone	ns	0.1168
Colon vs. Bone	ns	0.5057

Table S6. Tissue water content

Table S6. Tissue water content as described by Ali and Saber.[1]

Tissue type	Muscle	Fat	Spleen	Lung	Liver	Kidney	Bone
Water content (%)	73-78	5-20	76-81	80-83	73-77	78.79	44-45

Table S7. Total Al tissue content

Table S7. *The average lung was found to have a ten-times higher absolute Al amount than liver, the tissue with the second highest absolute Al content. <LOQ = below limit of quantification; NA = not available*

	Total Al in tissue / mg									
patient	1	2	3	4	5	6	7	8	Mean	SD
Lung	19.5	NA	20.3	7.94	2.36	1.44	0.59	NA	8.68	9.05
Heart	2.77	NA	<LOQ	0.08	0.06	0.08	0.10	0.09	0.45	1.02
Kidney	0.04	NA	<LOQ	<LOQ	0.04	0.06	0.04	0.05	0.03	0.02
Spleen	0.07	NA	0.06	0.11	0.02	0.01	<LOQ	0.04	0.04	0.04
Liver	0.80	NA	0.56	2.10	0.31	0.27	1.11	0.54	0.81	0.64

Figure S1. Lumogallion and H&E staining of patient 2

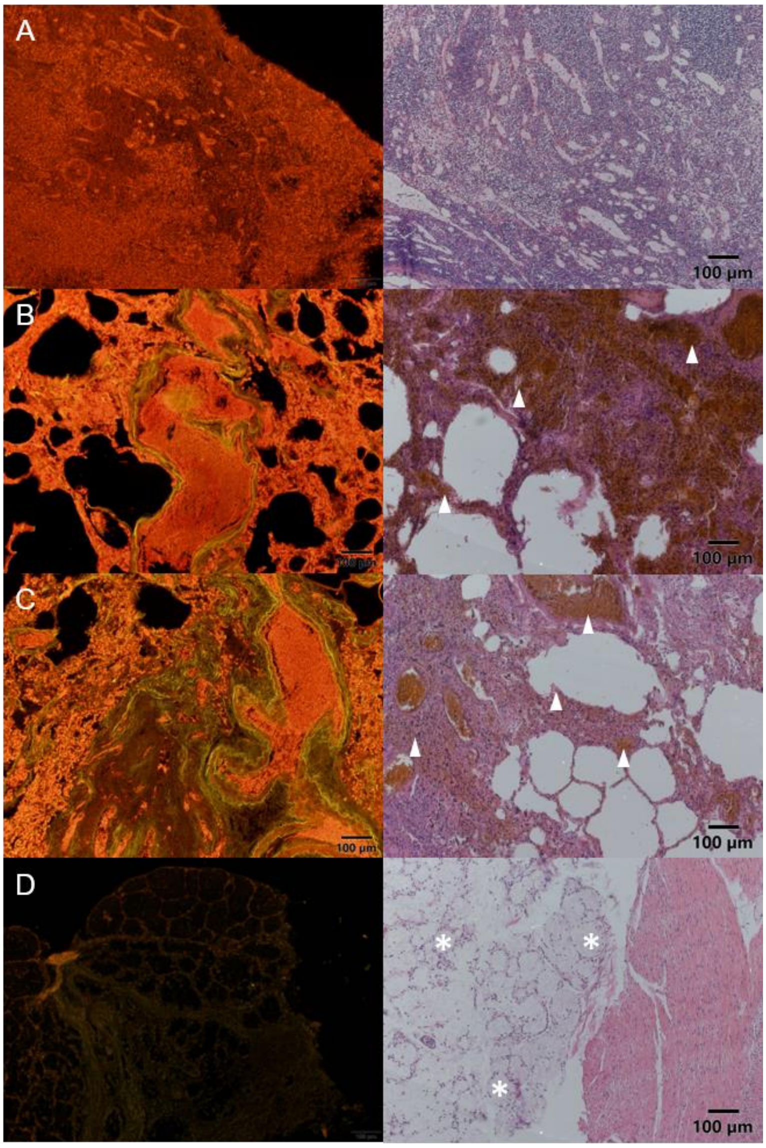

Figure S1. *Lumogallion (left column, red Al-specific fluorescence) and H&E images (right column) of hilar lymph node (A), upper lobe of the lung (B), lower lobe of the lung (C), and duodenum (D) are shown for patient 2. Erythrocytes are stained red using H&E (indicated with triangles) and are found inside blood vessels as well as within the tissue of upper and lower lobe of the lung. Like in patient 4 (see Figure 1), the location of erythrocytes and Al overlap, showing that Al is bound in erythrocytes in lung tissue. Brunner's glands were identified with H&E staining and are the only region of duodenum where Al signals are found (indicated with asterisks).*

Figure S2. Quarterly averages of Al in PM10 between 2014-2020

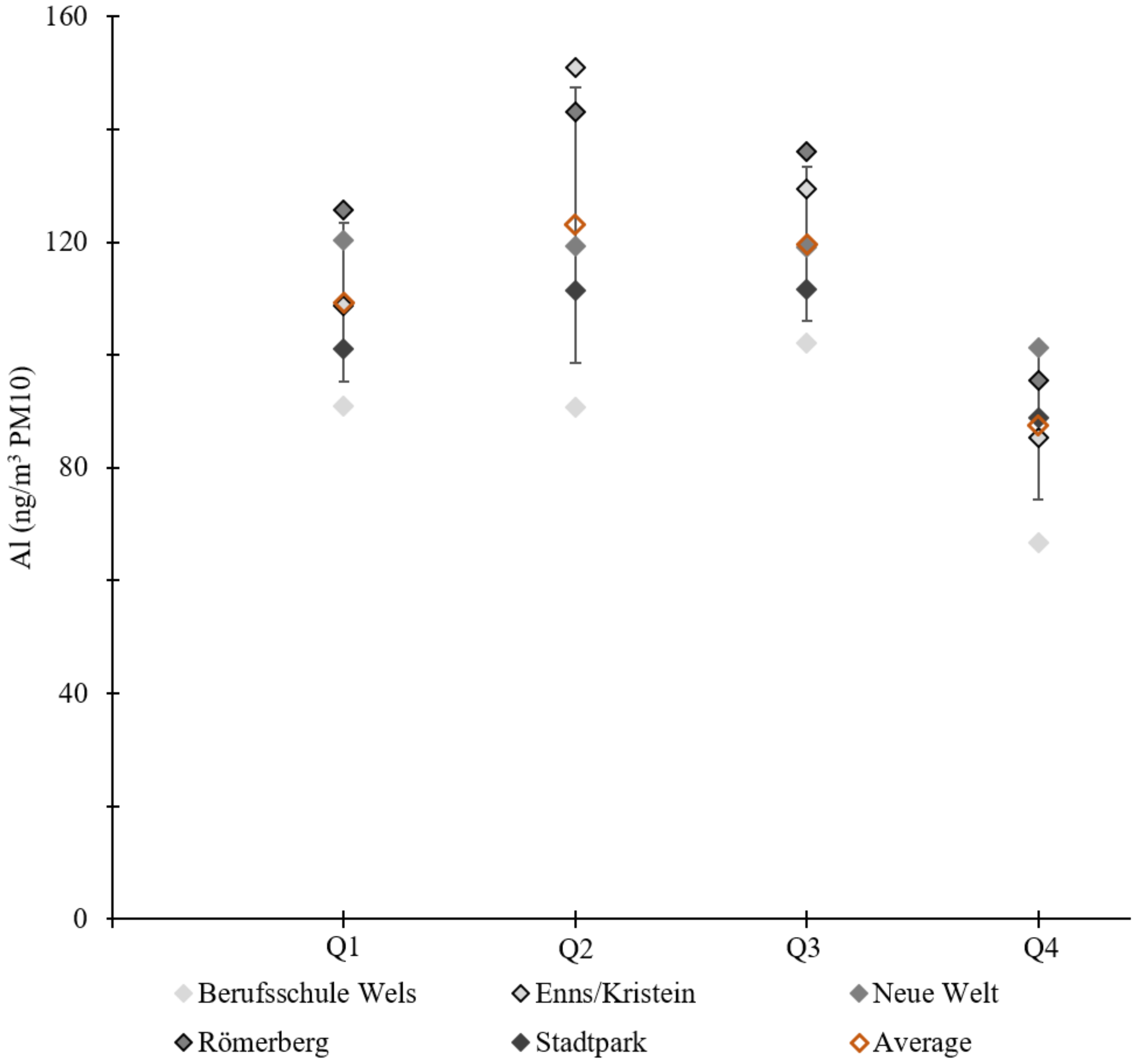

Figure S2. *Quarterly averages of Al in PM10 (in ng/m^3) were calculated for Römerberg, Enns/Kristein, Stadtpark and Neue Welt for 2014-2020, while data from 2015-2020 was used for Berufsschule Wels. The average of all quarterly averages of these locations of all years was calculated. Q1: January 1st - March 31st; Q2: April 1st - June 30th; Q3: July 1st - September 30th; Q4: October 1st - December 31st*

Supplementary References

(1) Ali, Y. E. M.; Alanaz, A. G. Temperatures Variation in Different Human Tissues according to Blood Flow Coefficient. *Int. J. Comput. Appl. Technol.* **2018**, *180* (28), 10–14.
